# Conversion Therapy for Advanced Pancreatic Cancer: The Case Series and Literature Review

**DOI:** 10.3389/fphar.2020.579239

**Published:** 2020-10-06

**Authors:** Mingxing Wang, Yunyun Xu, Min Yang, Dingyi Jiang, Yunwang Chen, Jiahong Jiang, Zheling Chen, Liu Yang, Dongsheng Huang

**Affiliations:** ^1^Department of Medical Oncology, Zhejiang Provincial People’s Hospital, People’s Hospital of Hangzhou Medical College, Hangzhou, China; ^2^Graduate School of Clinical Medicine, Bengbu Medical College, Bengbu, China; ^3^Department of Gastrointestinal and Pancreatic Surgery, Zhejiang Provincial People’s Hospital, People’s Hospital of Hangzhou Medical College, Hangzhou, China; ^4^The Qingdao University Medical College, Qingdao, China; ^5^Key Laboratory of Tumor Molecular Diagnosis and Individualized Medicine of Zhejiang Province, Zhejiang Provincial People’s Hospital, People’s Hospital of Hangzhou Medical College, Hangzhou, China

**Keywords:** conversion therapy, chemotherapy, advanced pancreatic cancer, surgery, positron emission tomography-computed tomography (PET-CT)

## Abstract

**Background:**

Pancreatic cancer has a high incidence and mortality. Most patients are in an advanced stage at the time of initial diagnosis and cannot be cured by a single surgery. The ASCO clinical practice guideline emphasized the overall management and multidisciplinary comprehensive treatment which put forward the concept of conversion therapy. In this paper, the real-world observation and study were carried out to explore the conversion effect of chemotherapy in patients with advanced pancreatic cancer and their long-term survival.

**Methods:**

The subjects of this study are advanced pancreatic cancer patients who visited the oncology department of Zhejiang Provincial People’s Hospital from 2015 to 2019. Collected and summarized the cases, and selected 5 representative patients for analysis, all of them received standard treatment (FOLFIRINOX, AS, AG, or GS). The progress, clinical evaluation, adverse reactions, and prognosis of these patients after conversion therapy were analyzed and discussed in conjunction with relevant literature.

**Results:**

Five patients with advanced pancreatic cancer received conversion therapy with an average survival time of 29.8 months, two of them received surgical treatment, and postoperative evaluations were pathological complete response (pCR). The tolerance of chemotherapy was good in five patients, and no serious adverse reactions of grade 3 or 4 occurred.

**Conclusion:**

Conversion therapy for patients with advanced pancreatic cancer strives for surgical opportunities of radical resection, prolongs survival and improves quality of life.

## Introduction

Pancreatic cancer is one of the most malignant neoplasms of the digestive system. The incidence and mortality have been increasing in recent years worldwide. It is estimated that pancreatic cancer will become the second leading cause of cancer death in the United States by 2030 (Rahib et al., 2014). At present, pancreatic cancer is the seventh most common malignant tumor and the sixth most deadly in China (Jia et al., 2018). Due to the concealed early clinical manifestations, most patients were diagnosed with locally advanced or advanced stage at the initial diagnosis, which often lost the opportunity of radical surgery (Tempero et al., 2012; Ducreux et al., 2015). The five-year survival rates of advanced pancreatic cancer are only 2% to 6%, and the median survival time is 3 to 11 months (Rochefort et al., 2019). Comprehensive treatment is the key to improve the prognosis of pancreatic cancer.

Currently, the clinical treatments for advanced pancreatic cancer are limited. The ASCO clinical practice guideline-recommended FOLFIRINOX or gemcitabine plus albumin-bound paclitaxel as the first-line palliative chemotherapy for patients with ECOG PS 0 to 1, and gemcitabine alone for patients with ECOG PS 2 or with a comorbidity profile that precludes other regimens (Sohal et al., 2016; Sohal et al., 2018). In recent years, targeted therapy, immunotherapy, biotherapy, and so on have broadened the prospects for the treatment of advanced pancreatic cancer (Ko, 2015; Krantz et al., 2017). Such new therapies seemed to improve the survival to some extent, but the efficacy achieved so far is limited. It is urgent to explore new methods to treat advanced pancreatic cancer. Therefore, the concept of comprehensive treatment needs to be proposed here.

The concept of conversion therapy is an integral part of comprehensive treatment. For advanced pancreatic cancer, there is no chance of surgery at the time of initial diagnosis. However, conversion therapy can turn this impossible situation into a possible (Frigerio et al., 2017; Furuse et al., 2018). The purpose of conversion therapy is to reduce the size of the tumor using radiotherapy, chemotherapy, or targeted therapy. After several cycles of conversion therapy, the clinicians could reassess the lesion. At this time, it is possible for the lesion to obtain an opportunity for surgery and achieve R0 resection, and the survival time of the patient can be further extended. The improvement of the conversion rate depends on the standardized management of patients, the multidisciplinary diagnosis and treatment mode, and the tolerance of patients to treatment. The clinical efficacy of conversion therapy for advanced pancreatic cancer has not been fully studied. This paper mainly discusses the efficacy and prognosis of conversion therapy for advanced pancreatic cancer through case reports and literature review, to provide effective treatment options for patients with advanced pancreatic cancer.

## Case Reports

### Case 1

**Figure 1:**
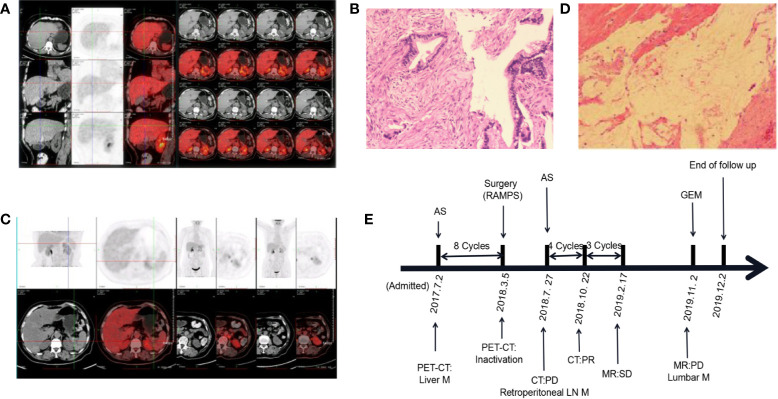
Changes in the condition after conversion therapy. **(A)** Positron emission tomography-computed tomography (PET-CT) showed increased FDG metabolism in the tail of the pancreas and the right lobe of the liver. **(B)** The histopathology of the pancreatic mass suggests adenocarcinoma. **(C)** PET-CT revealed a suppressed pancreatic tumor. **(D)** No malignant tumor cells were observed under the microscope. **(E)** The treatment process from the initial diagnosis to the end of follow-up. MR, magnetic resonance; M, metastasis; LN, lymph node; AS, albumin-bound paclitaxel plus S-1; GEM, gemcitabine; PD, progressive disease; PR, partial response; SD, stable disease.

A 69-year-old female presented to a local hospital with upper abdominal pain combined with radiation pain in the lower back. Abdominal computed tomography (CT) suggested that there was a mass located in the tail of the pancreas. Then she was referred to our hospital in July 2017. Serum carbohydrate antigen 19-9(CA19-9) level was 5,362 U/ml. Positron emission tomography-computed tomography (PET-CT) revealed that low-density masses located in the tail of the pancreas and the right lobe of the liver, with unevenly increased FDG metabolism, several lymph node shadows were found in the abdominal aorta and the left common iliac artery of paravertebral (**Figure 1A**). The pathological result of laparoscopic biopsy suggested the adenocarcinoma (**Figure 1B**). The patient was diagnosed with pancreatic ductal adenocarcinoma, and the clinical stage of T4N1M1. According to the multidisciplinary treatment (MDT) discussion, the patient did not indicate operation. The AS regimen [albumin-bound paclitaxel 200 mg/m^2^ on days 1 and 8, S-1 60 mg twice daily on days 1–14, every 3 weeks] was selected for conversion therapy in July 2017. After eight cycles of AS regimen, serum CA19-9 dropped to the normal range. CT showed that the pancreatic tumor continued to shrink, and PET-CT revealed that the pancreatic tumor was suppressed (**Figure 1C**). Therefore, radical antegrade modular pancreatosplenectomy (RAMPS) was performed in March 2018. No cancer cells were found in the primary lesion and dissected lymph nodes (**Figure 1D**). The pathological stage was pT3N0M0, and the postoperative evaluation was pathological complete response (pCR). In July 2018, retroperitoneal lymph node metastasis occurred. Then the patient was given an AS regimen for palliative chemotherapy again. Until the end of the follow-up, the overall survival (OS) reached 31 months (**Figure 1E**).

### Case 2

A 74-year-old male was admitted to the hospital in June 2016 with upper abdominal pain for 3 months. Serum CA19-9 level was 9.3 U/ml. Enhanced abdominal CT revealed a 41 mm*22 mm mass in the pancreatic body. The celiac artery (CA), superior mesenteric artery (SMA), and splenic artery (SA) showed encasement by direct tumor invasion and multiple slightly low-density nodules were found in the liver. An endoscopic ultrasound-guided biopsy of the pancreatic tail mass was arranged, and histology confirmed pancreatic adenocarcinoma. Based on these clinical findings, the patient was diagnosed with pancreatic ductal adenocarcinoma and the clinical stage of T4N1M1. Subsequently, the patient began to receive 9 cycles of GS regimen [gemcitabine 1,500 mg/m2 on days 1 and 8, S-1 60 mg twice daily on days 1–14, every 3 weeks]. After seven cycles, the pancreatic tumor size decreased to 21*12mm, and the efficacy was evaluated as a partial response (PR). Then, he was treated with S-1 alone to maintain chemotherapy, but the reexamination after two cycles suggested that the disease progressed. Thus, at the end of March 2017, the GS regimen was chosen again. During seven cycles of GS regimen, the size of the pancreatic tumor did not change much, and the efficacy evaluation was stable disease (SD). In June 2018, the patient underwent pancreatic cancer surgery and achieved R0 resection. PET-CT showed that the liver metastasis was completely inactivated. The postoperative stage was T3N1M0, and the evaluation was pCR. The patient received capecitabine monotherapy regularly after surgery and is still alive. Up to the end of follow-up, the OS was 46 months.

### Case 3

A 69-year-old female was admitted to the hospital at the end of May 2017 with upper abdominal discomfort for 3months. PET-CT revealed that the neck and body of the pancreas were significantly thickened, and the fluorodeoxyglucose (FDG) metabolism was abnormally increased. Multiple metastases were found in the liver, abdominal cavity, pelvic cavity, and retroperitoneal lymph node. Pancreatic biopsy revealed adenocarcinoma. The patient was diagnosed with pancreatic ductal adenocarcinoma, and the clinical stage of T4N1M1. In June 2017, the patient began to receive an AG regimen [albumin-bound paclitaxel 200 mg/m2 on days 1, 8, 15; gemcitabine 1,400 mg/m2 on days 1 and 8, 15, every 4 weeks]. The curative effect was evaluated as SD after four cycles. However, the disease progressed after seven cycles, abdominal CT showed a 40*22mm solid-cystic lesion in the pelvic cavity. Consequently, the patient switched to the FOLFIRINOX regimen [oxaliplatin 85 mg/m^2^; irinotecan 180 mg/m^2^; 5-fluorouracil 400 mg/m^2^; 5-fluorouracil 2400 mg/m^2^] in December 2017. After 8 cycles, CT showed that the size of pelvic lesion decreased to 28*12mm, the efficacy was PR. After 18 cycles, S-1 was given oral monotherapy for maintenance. In March 2019, the disease progressed again, the pelvic metastases increased significantly compared with January 2019 (103*95mm VS 55*77mm). After that, the patient lost to follow up and the OS was 23 months.

### Case 4

A 61-year-old male was admitted to the hospital in April 2016 with constipation. Enhanced hepatobiliary magnetic resonance (MR) suggested a cystic solid mass located in the body and tail of the pancreas with multiple abnormal enhancement foci in the liver and spine. The clinical diagnosis was pancreatic ductal adenocarcinoma with liver metastasis, the clinical stage of T4N0M1. At the end of April 2016, the patient was treated with 3 cycles of AG regimen [albumin-bound paclitaxel 200 mg/m2 on days 1, 8, 15; gemcitabine 1,700 mg/m2 on days 1 and 8,15, every 4 weeks]. After 2 cycles, MR showed that the largest mass in the liver was smaller than the initial one (26*27mm VS 30*40mm), the efficacy was PR. Subsequently, the patient received a GS regimen [gemcitabine 1,700 mg/m2 on days 1 and 8, S-1 60 mg twice daily on days 1–14, every 3 weeks]. After 2 cycles, the size of the largest lesion in the liver decreased to 25*20mm. However, the patient was intolerant, so the patient switched to gemcitabine monotherapy in September 2016. After seven cycles, the disease progressed. Then the patient tried several regimens, including gemcitabine plus capecitabine, Oxaliplatin plus capecitabine, and S-1 monotherapy. In September 2017, the secondary orbital metastasis of pancreatic cancer was diagnosed. The orbital metastasis was removed with gamma knife, and treated with two cycles of AS regimen. In November 2017, the patient was admitted to the hospital due to malignant pleural effusion, and received intraluminal injection of cisplatin and symptomatic support treatment. In December 2017, the patient and his family members asked to be discharged, the OS was 20 months.

### Case 5

**Figure 2:**
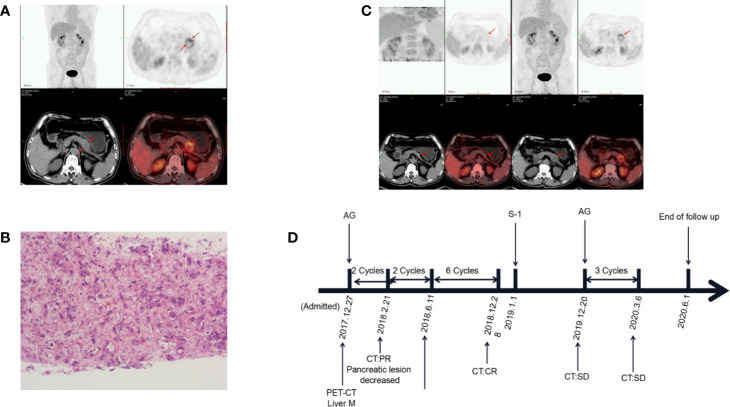
Changes in the condition after conversion therapy. **(A)** Fluorodeoxyglucose (FDG) metabolism increased in the body of the pancreas and liver. **(B)** The histopathology of the liver mass suggests adenocarcinoma. **(C)** Positron emission tomography-computed tomography (PET-CT) showed that tumors were completely inactivated. **(D)** The treatment process from the initial diagnosis to the end of follow-up. M, metastasis; PR, partial response; CR, complete response; SD, stable disease; AG, albumin-bound paclitaxel plus gemcitabine.

A 63-year-old male patient was admitted to the hospital at the end of December 2017 with epigastric distension and pain. Serum CA19-9 level was 417.0 U/ml. Abdominal CT revealed a 36*28mm mass between the body and tail of the pancreas, involving splenic artery and vein, and multiple hypodense lesions in the liver. PET-CT showed that FDG metabolism was increased in the density focus of soft tissue in the body of the pancreas, multiple intrahepatic lesions, and some retroperitoneal lymph nodes (**Figure 2A**). The pathological biopsy of intrahepatic masses suggested poorly differentiated adenocarcinoma (**Figure 2B**). The patient was diagnosed with pancreatic ductal adenocarcinoma, the clinical stage of T4N1M1. Subsequently, the patient was treated with AG regimen [albumin-bound paclitaxel 200 mg/m2 on days 1, 8, 15; gemcitabine 1,600 mg/m2 on days 1 and 8, 15, every 4 weeks] in January 2018. After 2 cycles, the tumor marker CA19-9 was reduced to 29.8 U/ml. CT showed that the size of the pancreatic lump decreased to 17*19mm, the liver lesions were unclear. After four cycles, PET-CT showed that there was no significant increase in FDG metabolism in the pancreas and liver, so tumors were considered to be completely inactivated (**Figure 2C**). After seven cycles, pancreatic and liver lesions were unclear after reexamination of CT scan. The curative effect evaluation was complete response (CR). In December 2019, serum CA19-9 level was 164.1 U/ml and gradually increased. Therefore, the patient switched to the original AG regimen to continue chemotherapy in December 2019, and serum CA19-9 dropped to 67.8 U/ml after one cycle. The patient is still undergoing an AG regimen for chemotherapy. The OS at the end of this follow-up was 29 months (**Figure 2D**).

## Discussion

The early detection rate of pancreatic cancer is low and it is easy to metastasize, which brings a great challenge to the treatment of pancreatic cancer (Andersson, 2017; Moore and Donahue, 2019). With the continuous updating of clinical research data, the diagnosis and treatment of advanced pancreatic cancer are also continuously improved. Before the emergence of gemcitabine, fluorouracil was the cornerstone of palliative treatment of advanced pancreatic cancer, then gemcitabine replaced fluorouracil as the treatment standard for advanced pancreatic cancer (Thota et al., 2014; Vaccaro et al., 2015). In the latest guidance, FOLFIRINOX and gemcitabine plus albumin-bound paclitaxel are now considered as standard first-line treatment for advanced pancreatic cancer. The improvement of chemotherapy regimens has prolonged the survival time of patients with advanced pancreatic cancer, but the effect of chemotherapy alone is still limited. Does it mean a total loss of surgery for unresectable pancreatic cancer? Providing these patients with surgical opportunities through comprehensive treatment plays an increasingly important role in the recent clinical practice (Furuse et al., 2018). After a period of conversion therapy, the primary and metastatic lesions in patients with metastatic pancreatic cancer who are sensitive to chemotherapy will gradually shrink or even reach R0 resection (Sakuraba et al., 2013; Maeda et al., 2014; Koga et al., 2014; Makino et al., 2015). Conversion therapy has won the chance of radical resection for patients with advanced pancreatic cancer and is expected to provide a new treatment strategy for patients with advanced pancreatic cancer. It has been found that conversion therapy can improve the prognosis and increase the survival rate of patients with initially unresectable pancreatic cancer who respond well to non-surgical treatment (Kato et al., 2011; Asano et al., 2018). The purpose of conversion therapy is to convert unresectable tumors into resectable tumors, while the purpose of neoadjuvant therapy is to expand the surgical effects of resectable tumors. This is the essential difference between them.

These five patients with stage IV pancreatic cancer all responded well to conversion therapy, and the average overall survival was 29.8 months. The longest OS time was 46 months and the survival interval was 20 to 46 months. Due to the better physical fitness score during the conversion treatment, the corresponding chemotherapy tolerance was also better. No serious adverse reactions occurred, and the long-term survival quality of life was also well. Reviewing previous literature, chemotherapy, targeted therapy, and immunotherapy have made breakthroughs in the treatment of advanced pancreatic cancer, and the overall survival of patients has been extended to a certain extent but still not more than 1 year (Ghosn et al., 2014; Sclafani et al., 2015; O’Hayer and Brody, 2016; Verdaguer et al., 2018). The average overall survival of these five patients was far more than one year, and no serious grade 3 or 4 adverse reactions occurred. Two patients achieved R0 resection and pathological complete response, and the other three patients achieved clinical partial response in imaging. Thus, patients with advanced pancreatic cancer should actively undergo conversion therapy and strive for the opportunity of radical surgical resection. Even if some patients do not undergo surgery in the end, from the perspective of three cases with an average survival time of 24 months without surgery, the benefits of conversion therapy for patients can also be reflected in the extension of overall survival.

During conversion therapy, the patients’ condition should be evaluated regularly to adjust the treatment plan in time. CT is a common tool to measure the tumor size of pancreatic cancer in the clinic. But for lesions less than two centimeters, the sensitivity of CT (40%) is not as high as that of PET-CT (100%) (Okano et al., 2011). PET-CT can evaluate the metabolic activity of tumors through maximal standardized uptake value (SUVmax), which plays an important role in the subsequent selection of surgical treatment (Wakabayashi et al., 2008). Besides, serum CA19-9 is the only biomarker of pancreatic cancer approved by the Food and Drug Administration (FDA) (Fong and Winter, 2012). The change of serum CA19-9 level reflects the response of patients to treatment, helping to assess disease progression and tumor metastasis, which is of great significance to the prognosis and recurrence of the patients (Willett et al., 1996; Fong and Winter, 2012; Scarà et al., 2015). The SUVmax combined with the CA19-9 level can significantly improve the sensitivity and accuracy of pancreatic cancer detection (Sun et al., 2015). Therefore, patients with advanced pancreatic cancer should regularly undergo PET-CT examination and tumor marker CA19-9 detection during the conversion treatment process, and capture the appropriate time to decide whether to continue chemotherapy or to perform surgical treatment. In this study, some patients did not choose surgery because of their reasons, but researchers all conducted timely communication and notification and carried out comprehensive treatment for patients based on respecting patients’ wishes.

This study also has some limitations. This study was a retrospective single-center clinical study with a small sample size and lack of concurrent controlled analysis. In the future, we expect to design clinical studies with larger sample sizes to provide more sufficient clinical evidence for the conclusions of this study.

## Conclusion

In this case series report, all five patients received conversion therapy and obtain the average 29.8 months overall survival time and the longest was 46 months. In this study, we thought that PET-CT scan after conversion therapy played an important role in the lesion activity assessment. The patient survival benefits are reflected in pCR, OS, and R0 rates. For patients with advanced pancreatic cancer, conversion therapy on the one hand provides patients with the opportunity to undergo surgery and prolongs their survival; on the other hand, their long-term quality of life improves.

## Data Availability Statement

The original contributions presented in the study are included in the article/supplementary material; further inquiries can be directed to the corresponding authors.

## Ethics Statement

The studies involving human participants were reviewed and approved by: The ethics committee of the Zhejiang Provincial People’s Hospital. The patients/participants provided their written informed consent to participate in this study. Written informed consent was obtained from the individual(s) for the publication of any potentially identifiable images or data included in this article.

## Author Contributions

LY, DH, and ZC contributed to the conception of the study. MW integrated all information and wrote the manuscript. LY, ZC, and JJ provided critical guidance, revisions for MW throughout the writing process. YX, MY, DJ, and YC compiled information and revised the manuscript. All authors contributed to the article and approved the submitted version.

## Funding

This work is supported by the Key Foundation of Science Technology Department of Zhejiang Province (No.2015C03030), the National Natural Science Foundation of China (No.81772575, No. 81802623).

## Conflict of Interest

The authors declare that the research was conducted in the absence of any commercial or financial relationships that could be construed as a potential conflict of interest.
